# Melody transcatheter pulmonary valve replacement: a single-center case series in Southeast Asia

**DOI:** 10.1186/s12872-024-03919-7

**Published:** 2024-06-13

**Authors:** Marin Satawiriya, Mann Chandavimol, Alisa Limsuwan

**Affiliations:** 1 Division of Pediatric Cardiology, Department of Pediatrics, Faculty of Medicine Ramathibodi Hospital, Mahidol University, 270 Rama 6 Rd, Rachathewi, Bangkok, 10400 Thailand; 2 Division of Cardiology, Department of Internal Medicine, Faculty of Medicine Ramathibodi Hospital, Mahidol University, Bangkok, Thailand

**Keywords:** Transcatheter pulmonary valve replacement, Melody valve, Intervention

## Abstract

**Background:**

Studies of transcatheter pulmonary valve replacement (TPVR) with the Melody valve have demonstrated good clinical and hemodynamic outcomes. Our study analyzes the midterm clinical and hemodynamic outcomes for patients who underwent Melody valve implantation in Southeast Asia.

**Methods:**

Patients with circumferential conduits or bioprosthetic valves and experiencing post-operative right ventricular outflow tract (RVOT) dysfunction were recruited for Melody TPVR.

**Results:**

Our cohort (*n* = 14) was evenly divided between pediatric and adult patients. The median age was 19 years (8–38 years), a male-to-female ratio of 6:1 with a median follow-up period of 48 months (16–79 months), and the smallest patient was an 8-year-old boy weighing 18 kg. All TPVR procedures were uneventful and successful with no immediate mortality or conduit rupture. The primary implant indication was combined stenosis and regurgitation. The average conduit diameter was 21 ± 2.3 mm. Concomitant pre-stenting was done in 71.4% of the patients without Melody valve stent fractures (MSFs). Implanted valve size included 22-mm (64.3%), 20-mm (14.3%), and 18-mm (21.4%). After TPVR, the mean gradient across the RVOT was significantly reduced from 41 mmHg (10–48 mmHg) to 16 mmHg (6–35 mmHg) at discharge, *p* < *0.01.* Late follow-up infective endocarditis (IE) was diagnosed in 2 patients (14.3%). Overall freedom from IE was 86% at 79 months follow-up. Three patients (21.4%) developed progressive RVOT gradients.

**Conclusion:**

For patients in Southeast Asia with RVOT dysfunction, Melody TPVR outcomes are similar to those reported for patients in the US in terms of hemodynamic and clinical improvements. A pre-stenting strategy was adopted and no MSFs were observed. Post-implantation residual stenosis and progressive stenosis of the RVOT require long term monitoring and reintervention. Lastly, IE remained a concern despite vigorous prevention and peri-procedural bacterial endocarditis prophylaxis.

## Background

The incidence of congenital heart disease worldwide is generally reported to be 8 in 1000 live births, with the prevalence of cardiac anomaly at birth highest in Asia (9.3/1000 live births) [1]. Interestingly, right-sided congenital cardiac anomalies have been reported to be more prevalent in Asians than in Caucasians [1, 2]. To treat right-sided heart obstruction, significant numbers of patients have undergone surgical repair of the right ventricular outflow tract (RVOT), with some hemodynamic sequelae. The postoperative RVOT dysfunction, in terms of pulmonary obstruction or regurgitation, requires further intervention with either surgical or transcatheter procedures. In 2000, Phillip Bonhoeffer and colleagues reported the first successful transcatheter pulmonary valve replacement (TPVR) using a bovine jugular venous valve in a platinum iridium stent frame [3–5], representing a significant milestone for RVOT transcatheter intervention. The successor to that valve was the Melody transcatheter pulmonary valve (Medtronic, Inc., Minneapolis, MN, USA), and a series of studies have since described the clinical improvements and good hemodynamic outcomes associated with the valve [4, 6–14]. In 2010, the Melody valve received approval under a Humanitarian Device Exemption by the US Food and Drug Administration, followed by Pre-Market Approval in 2015 for implantation in the circumferential RVOT conduit.

The introduction of the Melody valve facilitated the successful implementation of TPVR in Southeast Asia during the early-2010s. Ramathibodi Hospital, Mahidol University, emerged as the first certified solo center for TPVR in the region in 2016, where significant experience has been gained in implanting the Melody valve and other TPVR procedures. The adoption of TPVR in this region has been delayed due to its high cost and lack of coverage by existing healthcare schemes. To address this challenge, we initiated a TPVR project subsidized by the Ramathibodi Foundation through donated funds. This endeavor serves as an initial step towards leveraging TPVR technology, enabling us to gain experience and confidence. In this report, we present midterm clinical and hemodynamic outcomes for the patients initially treated in this region, underscoring the feasibility and effectiveness of our approach.

## Materials & methods

### Patients and procedure

The Melody valve implantation protocol requires TPVR candidates to have circumferential surgical conduit anastomosis between the right ventricle [15] and the pulmonary artery (PA) that is dysfunctional, and/or a failed bioprosthetic valve (BPV). Therefore, prior to enrollment for TPVR evaluation, we identified patients that had previously implanted circumferential RVOT conduits and/or BPVs from a group of patients with repaired Tetralogy of Fallot and RVOT reconstruction. This study received ethical approval from the Ethical Clearance Committee on Human Rights at the Faculty of Medicine Ramathibodi Hospital.

This was a retrospective cohort study, a nonrandomized single-center study designed to follow patients for 10 years after TPVR, with predetermined windows of follow-up. We selectively enrolled patients with RVOT dysfunction after circumferential RV-to-PA conduit anastomosis or failed BPV. All previous clinical data were retrospectively reviewed, including previous surgeries, clinical status, and cardiac evaluation with echocardiogram or magnetic resonance imaging (MRI). The initial criteria for Melody valve implantation included conduit diameter of less than 25 mm due to the limited size of Melody valves available; the largest valve has an inner diameter of 22 mm which can be expanded to 24.5 mm by high pressure balloon.

Our indications for Melody valve implantation were symptomatic patients with conduit dysfunction or failed BPV, or asymptomatic patients with significant pulmonary stenosis (PS) with right ventricular systolic pressure of more than 60% of systemic pressure and/or significant pulmonary regurgitation (PR) [16] with regurgitation fraction > 25% and significant dilated right ventricle (RV) (RV end-diastolic volume index > 150 ml/m^2^) [17].

Patients meeting these criteria underwent cardiac catheterization under general anesthesia, and right and left heart hemodynamics were first evaluated. An initial angiogram was obtained to measure the diameters of the RVOT, conduit, and PA. Then a sizing balloon was advanced and inflated to measure the stretched diameter of the RVOT and conduit. Subsequently, coronary compression was tested using a high-pressure balloon (Atlas Gold PTA) to occlude the RVOT, accompanied by the aortic root angiogram that best profiled the relationship of the coronary arteries to the RVOT. Patients with coronary compression or aortic valve regurgitation were excluded from implantation. Significant RVOT obstruction with RV systolic pressure higher than 60% of the systemic pressure and/or significant pulmonary regurgitation with RV dilation were the main indications for Melody valve implantation.

Our regional protocol for Melody valve implantation recommended placing a new pre-stent in a stentless conduit concomitantly with TPVR. We used the covered Cheatham Platinum (CP) stent. The valve was delivered using the Melody Ensemble delivery system through venous sheath (via either femoral vein or internal jugular vein). All patients received heparin to maintain an activated clotting time > 250 s and antibiotic prophylaxis during and after the procedure. After implantation, measurements of hemodynamics and PA angiography were obtained. Post-dilation of the valve immediately after deployment was performed at the discretion of the operator.

### Follow-up

Outcomes of the procedure, including immediate complications during TPVR, were monitored over time. Follow-up for the first year consisted of clinical history and physical examination, echocardiogram, and chest film every 6 months. Thereafter, this information was collected during annual follow-up visits. Radiographic studies were performed to detect Melody valve stent fractures (MSFs), particularly in patients without concomitant conduit pre-stenting. Cardiac MRI was conducted 12–36 months after TPVR.

### Statistical analysis

To describe procedural outcomes, data are presented as frequency (%) for categorical variables and as median (minimum–maximum) or mean ± standard deviation (SD) for continuous variables. The primary outcome was transcatheter pulmonary valve (TPV) reintervention; secondary outcomes included clinical status, Melody valve dysfunction (obstruction and/or regurgitation), MSF, endocarditis, and other TPV-related adverse events. TPV reinterventions were defined as dilation, TPV-in-TPV implantation, or explantation. RVOT gradient was presented as the mean Doppler gradient across the conduit or valve. Gradients were analyzed at preimplant, discharge, and follow-up, and compared using the paired t-test and Wilcoxon rank-sum test to determine the difference between pre- and post-procedural RVOT gradient.

## Results

### Patients and procedure

**Fig. 1:**
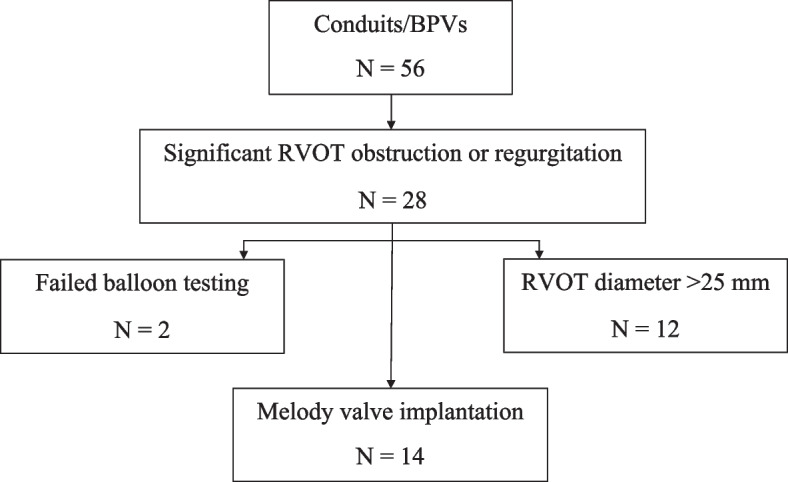
Patient flow. *BPV* Bioprosthetic valve, *RVOT* Right ventricular outflow tract, *TPVR* Transcatheter pulmonary valve replacement

From a group of 286 surgical RVOT reconstruction patients, we identified 56 patients that had circumferential RVOT conduit anastomosis or a BPV. Half of these patients (*n* = 28) had significant RVOT obstruction or regurgitation and underwent cardiac catheterization. However, 12 were excluded from valve implantation due to a conduit diameter that exceeded the 25 mm Melody valve limit. Additionally, two other patients developed aortic regurgitation or coronary compression during the balloon sizing test, and implantation was not attempted. The remaining 14 patients were eligible for TPVR with the Melody valve and constitute the study cohort (Fig. 1).

Demographic and baseline characteristics, underlying disease, and conduit-related information for these patients are summarized in Table 1. Our cohort was evenly divided between pediatric and adult (> 18 years) patients, with a median age of 19 years (range 8–38 years), and a male-to-female ratio of 6:1. The majority of conduits (71.4%) were homograft conduits. Our smallest patient underwent TPVR at the age of 8 years and weighed 18 kg. A median follow-up period after TPVR was 48 months (range 16–79 months). Primary implant indication was combined stenosis and regurgitation for 6 patients, significant RVOT stenosis for 5 patients, and significant PR for 3 patients.

**Table 1:** Baseline characteristics and conduit-related data for the cohort of implanted patients

	All patients (*N* = 14)	Pediatric patients (*N* = 7)	Adult patients (*N* = 7)
Age, years	19 (8, 38)	13 (8, 18)	23 (20, 38)
Male (%)	12 (85.7%)	6 (85.7%)	6 (85.7%)
Body weight, kg	50.5 (18, 60)	37 (18, 54)	53 (45, 60)
**Underlying disease, N (%)**			
TOF	7 (50%)	3 (42.8%)	4 (57.2%)
PAVSD	4 (28.6%)	3 (42.8%)	1 (14.3%)
DORV with PS	2 (14.3%)	0	2 (28.6%)
VSD with PS	1 (7.2%)	1 (14.4%)	0
Previous median sternotomy surgery, number of times	2 (1, 2)	2 (1, 2)	2 (1, 2)
Follow-up period, months	48 (16, 79)	48 (27, 79)	47 (16, 68)
**RVOT materials, N (%)**			
Homograft conduits	10 (71.4%)	6 (85.7%)	4 (57.2%)
Bioprosthetic valves/valved conduits	4 (28.6%)	1 (14.3%)	3 (42.8%)
**Indication for TPVR**			
Stenosis	5 (35.7%)	3 (42.8%)	2 (28.6%)
Regurgitation	3 (21.4%)	0	3 (42.8%)
Mixed	6 (42.9%)	4 (57.2%)	2 (28.6%)

Data are presented as median (range minimum, maximum), or number (percentage)

*DORV* Double outlet right ventricle, *kg* kilogram, *mm* millimeter, *PAVSD* Pulmonary atresia with ventricular septal defect, *PS* Pulmonary stenosis, *RVOT* Right ventricular outflow tract, *TOF* Tetralogy of Fallot, *TPVR* Transcatheter pulmonary valve replacement, *VSD* Ventricular septal defect

The average conduit diameter was 21 ± 2.3 mm. Concomitant pre-stenting was placed in 9 patients (64.3%) and 1(7.1%) patient had concomitant stenting utilizing the one-step technique to crimp a covered-stent over the Melody valve, which was then crimped onto the balloon catheter of the Melody Ensemble delivery system [18]. All of the stents used were covered stents. Implanted valve sizes included 22 mm (*n* = 9; 64.3%), 20 mm (*n* = 2; 14.3%), and 18 mm (*n* = 3; 21.4%) (Table 2).

**Table 2:** Melody TPVR procedural details

	All patients (*N* = 14)	Pediatric patients (*N* = 7)	Adult patients (*N* = 7)
Interval from conduit replacement to TPVR, years	7 (3, 15)	7 (3, 12)	7 (3, 15)
Diameter of the conduit or BPV, mm	21.1 ± 2.3	21 ± 2.4	21.3 ± 2.4
Narrowest angiographic diameter, mm	16.9 ± 2.1	15.4 ± 1	18.2 ± 2
Concomitant Prestent, N (%)	9 (64.3%)	5 (71.4%)	4 (57.2%)
Concomitants Stent (One-step technique)	1 (7.1%)	0	1 (14.3%)
**Size of implanted valve**			
18 mm	3 (21.4%)	2 (28.6%)	1 (14.3%)
20 mm	2 (14.3%)	2 (28.6%)	0 (0%)
22 mm	9 (64.3%)	3 (57.2%)	6 (85.7%)
**Mean Doppler RVOT gradient by echocardiogram, mmHg**			
Preimplantation	41 (10, 48)	44 (40, 48)	38 (10, 46)
Discharge	16 (6, 35)	16 (9, 35)	15 (6, 31)
**Length of stay, days**	8.5 ± 2.1	9.1 ± 2.1	7.8 ± 1.8

Data are presented as mean ± SD, median (range minimum, maximum), or number (percentage)

*BPV* Bioprosthetic valve, *mm* millimeter, *mmHg* millimeter of mercury, *RVOT* Right ventricular outflow tract, *TPVR* Transcatheter pulmonary valve replacement

The valve diameter was selected according to the patient’s conduit diameter and angiographic RVOT measurement. Most of the patients (*n* = 8;57.1%) had valves implanted with the similar diameter as that of the conduit while 3 patients had larger diameter valves and 3patients had valves implanted with diameters smaller than the conduit or the BPV due to the largest Melody valve size available at 22 mm in 2 patients with larger conduits (Table 3). The case of Patient 9 illustrates the reasoning for smaller diameter selection. This patient had a known diagnosis of pulmonary atresia with ventricular septal defect status post-Rastelli’s operation with a 23-mm pulmonary homograft, and during the initial balloon occlusion testing using a 22-mm Atlas Gold PTA balloon, he had compromised right coronary flow. The decision was made to designate the landing zone above the original valve of the conduit, and when retested with a smaller and shorter balloon (Atlas Gold PTA 20 mm), his coronary angiogram indicated patency and normal flow of the coronary artery. Therefore, a 20-mm Melody valve was selected to deploy with the Ensemble delivery system using a 20-mm balloon.

**Table 3:** Melody valve: individual patient data and indications for TPVR

	Diagnosis	Conduit or BPV diameter, mm		Indication	Melody Valve, mm	Pre-Implant mean Doppler gradient (mmHg)	Discharge mean Doppler gradient (mmHg)	Follow-up mean Doppler gradient (mmHg)	Hospital Adverse events	Follow-up Adverse events
**1**	PAVSD	Homograft	23	PS	22	40	10	8	Fever H/C: No growth	-
**2**	TOF	Perimount BPV	19	PS & mod PR	18	44	9	30	Fever H/C: No growth	Cardiac arrest: VT, VF with IE (2 years later)
**3**	PAVSD	Hancock BPV	18	PS	18	39	17	18	-	IE (5 years later)
**4**	PAVSD	Homograft	21	PS & mod PR	20	40	14	8	Fever H/C: No growth	-
**5**	DORV/PS	Valved conduit	20	PS & severe PR	22	38	6	30	Fever bacteremia, no IE	-
**6**	DORV/PS	Homograft	19	PS	22	44	31	28	Fever H/C: No growth	-
**7**	VSD, PS	Homograft	19	PS & severe PR	18	41	17	14	Fever H/C: No growth	-
**8**	TOF	Homograft	24	PS & mod PR	22	48	16	17	Fever H/C: No growth	-
**9**	PAVSD	Homograft	23	PS	20	45	23	30	-	-
**10**	TOF	Homograft	22	Severe PR	22	10	13	13	Fever H/C: No growth	-
**11**	TOF	Porcine heterograft	23	Severe PR	22	19	20	10	Fever H/C: No growth	-
**12**	TOF	Homograft	25	Mod PR	22	26	15	8	Fever H/C: No growth	-
**13**	TOF	Homograft	18	PS	22	45	35	36	Fever H/C: No growth	-
**14**	TOF	Homograft	22	PS & mod PR	22	46	9	13	Fever H/C: No growth	-

*BPV* Bioprosthetic valve, *DORV* Double outlet right ventricle, *H/C* Hemoculture, *IE* Infective endocarditis, *mm* millimeter, *mmHg* millimeter of mercury, *mod* moderate, *PAVSD* Pulmonary atresia with ventricular septal defect, *PR* Pulmonary regurgitation, *PS* Pulmonary stenosis, *TOF* Tetralogy of Fallot, *TPVR* Transcatheter pulmonary valve replacement, *VF* Ventricular fibrillation, *VSD* Ventricular septal defect, *VT* Ventricular tachycardia

All TPVR interventions were uneventful and successful with no immediate mortality or conduit rupture.

### Acute hemodynamic results

After TPVR with the Melody valve, a competently functioning pulmonic valve was verified in all patients. The discharge echocardiogram revealed no or trivial PR, which was a significant improvement in 9 patients; 4 of whom had moderate PR and 5 of whom had severe PR prior to valve implantation. The mean gradient across the RVOT was significantly reduced from 41 mmHg (range 10–48 mmHg) at baseline to 16 mmHg (range 6–35 mmHg) at discharge, *p* < *0.01* (Table 2). Prior to valve implantation, pediatric patients had a slightly higher mean pressure gradient (44 mmHg, range 40–48 mmHg) than adults (38 mmHg, range 10–46 mmHg), *p* = *0.05*. However, post-implantation, the mean gradient was not significantly different among pediatric (16 mmHg, range 9–35 mmHg) and adult groups (15 mmHg, range 6–31 mmHg), *p* = *0.65* (Table 2).

Among the 11 patients with RVOT obstruction, including both PS and mixed PS/PR, 4 exhibited an immediate peak-to-peak RVOT gradient exceeding 15 mmHg following Melody TPVR. Subsequent Doppler echocardiographic evaluations upon discharge revealed that two of these patients (Patients 2 and 4) demonstrated a mean gradient across the right ventricular outflow tract (RVOT) of less than 15 mmHg, whereas Patients 9 and 13 continued to exhibit residual gradients exceeding 15 mmHg. Both of them had severely calcified conduits, they continued to demonstrate moderate RVOT gradients at discharge despite undergoing vigorous balloon dilatation with high-pressure balloons during TPVR.

### Immediate post-procedure adverse events

Immediately postprocedure, the majority of patients (78.5%) had a fever without a specific source of infection or bacteremia and received intravenous antibiotic(s) (Table 3). Only 1 patient had *Aeromonas caviae* bacteremia, without evidence of infective endocarditis (IE), and was treated with a full course of antibiotics. All patients were discharged with a functioning Melody valve in place.

### Follow-up adverse events and hemodynamics

Our median follow-up duration was 48 months (range 16–79 months) with no mortality occurring during this time. At 2 years follow-up, one patient (Patient 2) experienced cardiac arrest due to ventricular arrhythmia-related to IE. His condition was managed medically prior to planning for explantation. He developed moderate RVOT stenosis during and after IE treatment (Table 3).

Late follow-up IE was diagnosed in 2 patients (Patient 2 and Patient 3; Table 3) at 2- and 5-year postimplantation, respectively. For both patients, the primary indication for TPVR was failed BPV. Our cohort data indicated that overall freedom from IE was 86% at 79 months follow-up.

Table 3 presents mean Doppler gradients for each patient at preimplant, discharge, and follow-up. Patients 6, 9 and 13 had heavily calcified conduits with severe conduits stenosis. Despite aggressive balloon angioplasty and the use of the largest Melody valves (diameter of 22 mm) in Patients 6 and 13, they had significant RVOT residual gradients > 20 mmHg at discharge while the Patient 6 had the peak-to-peak gradient of 13 mmHg immediately after valve deployment.

During follow-up, 3 patients (patients 2, 5, and 9) developed progressive RVOT gradients. As noted above, Patient 2 developed late follow-up RVOT obstruction related to IE. Patient 5 had progressive conduit stenosis. These 2 patients were closely monitored. Patient 9, who had a smaller diameter valve deployed above the original valve annulus of the conduit without pre-stenting to avoid the compromised right coronary flow, had residual RVOT obstruction and a mean gradient of 23 mmHg at discharge that gradually progressed during follow-up and required redilation of the Melody valve. The patients with progressive RVOT obstruction after TPVR had a preimplantation mean Doppler gradient of 44 mmHg (range 38–45 mmHg) while the patients without obstruction had a preimplantation gradient of 40 mmHg (range 10–48) (*p* = *0.69).* However, the mean discharge Doppler gradients between these 2 groups were not statistically significant different (9 mmHg, range 6–23 mmHg, vs 16 mmHg, range 9–35 mmHg), *p* = *0.28.*

### Transcatheter melody valve reintervention

In the case of Patient 9, as previously described, a reintervention involving dilation was conducted 24 months post-implantation, resulting in a decrease in the peak-to-peak gradient from 60 to 30 mmHg. Patient 13 underwent redilation of the valve and conduit 46 months after the initial procedure, leading to a reduction in the peak gradient from 36 to 24 mmHg.

## Discussion

Advancements in TPVR technique and the availability of comprehensive follow-up data have elevated TPVR as a primary treatment option for failed bioprosthetic pulmonary valves (BPV), circumferential RVOT conduit dysfunction, or native RVOT dysfunction [19]. In the Southeast Asia region, the introduction of the Melody valve began in the early 2010s. A significant obstacle to TPVR adoption in the region has been limited health coverage and financial support for innovative medical procedures. Although our center was initially approached to pioneer Melody TPVR in Thailand, securing sufficient financial support posed a challenge.

However, leveraging our experience with a significant number of RVOT reconstruction patients and with the financial backing from the Ramathibodi Foundation, we initiated our Melody TPVR project in 2015. This endeavor culminated in our center being recognized as the First Certified Solo Center for Melody TPVR in Southeast Asia and the Asia Pacific region (outside of Australia and New Zealand).

Acknowledging similar financial challenges faced by other countries in the region, the introduction of lower-cost TPVR valves, specifically the Venus P-valve in 2014, offered a viable alternative. Despite initially serving as a human trial with approximately two-thirds of the cost of the pre-market approval Melody valve, we opted for the Melody valve due to its established human trial data.

Our center has gained modest experience in performing TPVR with the Melody valve in a select group of patients, yielding satisfactory outcomes in managing RVOT conduit dysfunction and BPV failure. Following TPVR, patients in our study cohort experienced improvements in hemodynamic and clinical outcomes comparable to those reported in the US Investigational Device Exemption Trial (IDE) [7, 20], including a competently functioning pulmonary valve, significant reduction in mean Doppler RVOT gradient, and absence or trivial pulmonary regurgitation on discharge echocardiogram.

Our single-center experience indicated that one-fifth of patients who underwent surgical RVOT reconstruction had implanted circumferential conduits or bioprosthetic valves. In turn, half of these patients had dysfunctional conduits or failed bioprosthetic valves. The cost of TPVR using a commercially available device such as the Melody valve is higher than the cost of RVOT surgery but has lesser morbidity with no mortality. In the merit of cost-effectiveness ratio, we are able to convince and obtain the funding from our hospital foundation to provide TPVR using the Melody valve for this specific group of patients with minimal copayment. Therefore, the case selection was dedicated to ensuring the success of the immediate procedure.

In the SE Asia region, Melody TPVR protocol required candidates to have either a circumferential conduit or bioprosthetic valve with a diameter between 16 to 24 mm. The majority of the patients had type II (tubular) and type V (hourglass) RVOT morphology [21]. Preprocedural imaging to evaluate the RVOT and adjacent anatomy, particularly the coronary arteries, was intensively reviewed. Out of 16 candidates, 2 patients (12.5%) failed the coronary compression testing Among our cohort of 14 patients, the Melody valve has proven effective in the majority of cases, with no mortality observed and no urgent surgeries related to the Melody TPVR procedure. However, approximately one-fifth of patients have experienced progressive RVOT stenosis. This occurrence is likely associated with a conservative approach to treating calcified stenotic conduits, prompted by concerns of conduit rupture as a potential complication. Despite this challenge, our approach has ensured patient safety and minimized adverse outcomes.

In a recent update on TPVR [22], the indications for TPVR were reported to differ between age groups, with older adults more likely to present with PR, and children more likely to be referred due to conduit stenosis. In our study, the primary indication for implantation was combined obstruction and regurgitation (42.9%), whereas in the US IDE study, the primary indication was PR (53%) [7]. The upper limit of the cohort age range in our study was younger than that of the US study, which may have influenced our findings. Because most of our cohort had isolated RVOT stenosis or combined RVOT stenosis, particularly calcified conduit, the Melody diameter selected was similar to the conduit diameter. Expansion of RVOT conduits beyond their nominal diameter to accommodate a larger Melody diameter was accomplished in 3 patients. Our follow-up data indicated no MSF, valve misplacement, or valve embolization, with 71.4% of patients having concomitant stenting.

Initial regulatory trials for TPVR excluded smaller patients (< 30 kg) [3, 4, 15, 23–25], but improvements in TPVR technique and gains in technical experience have allowed TPVR to be extended to smaller (< 20 kg) and younger patients [23]. In our cohort, the smallest patient was an 8-year-old male weighing 18 kg, and he successfully underwent TPVR via the femoral vessels without vascular complication. Therefore, we consider TPVR in selected children under 20 kg to be feasible and safe when performed at experienced centers [15, 23, 25].

Postimplanation IE is well recognized as a potential Melody TPVR complication [13, 26–28]. Therefore, we emphasized both perioperative antibiotic prophylaxis and postoperative subacute bacterial endocarditis prophylaxis. Nevertheless, there were 2 cases of IE at 2 and 5 years after TPVR. Despite a full course of intravenous antibiotics and complete recovery, one patient developed progressive valve stenosis. Based on this experience, our study reports a 14% incidence of IE, which is within the range (3.2%-25%) reported by other studies [29]. The other contributing factor for IE could be the basic oral hygiene and dental care of the people in the region.

Our approach to treating RVOT stenosis using a high-pressure balloon diameter of not more than 110% of the conduit diameter was conservative, and three of our patients (Patient 6, 9, and 13), have significant residual conduit stenosis (mean Doppler gradient > 20 mmHg). Patient 13 had concomitant stenting using the one-step technique by crimping the covered stent over the Melody valve which was crimped onto the Melody Ensemble delivery system. Despite the use of the Melody diameter (22 mm) larger than the conduit diameter (18 mm), there was significant residual conduit stenosis. Immediately post-dilation of the Melody valve and covered stent after deployment with a 24-mm Atlas Gold balloon was performed without the elimination of residual stenosis. The concomitant one-step technique could potentially set the limit for maximum expansion for both the Melody and covered stent. Whether a more aggressive approach to treat RVOT stenosis with a larger balloon would prevent the postimplantation residual stenosis, an approach that involves the risk of conduit rupture, needs further study and a risk–benefit analysis.

Although 3 patients (Patient 2, 5, and 9) developed progressive RVOT obstruction after Melody TPVR, we could not define the association between the combinations of preimplant and discharge mean gradients and post-implantation RVOT stenosis. Patient 9 and 13 underwent transcatheter Melody valve reintervention due to progressive RVOT stenosis, with RV systolic pressure > 60% systemic pressure, a preimplantation mean gradient ≥ 35 mmHg and a postimplantation mean gradient > 20 mmHg. According to results obtained in the US IDE trial [7], these factors would place these patients at the highest risk for reintervention.

## Limitations

Our limited number of patients precluded meaningful comparison with data obtained in the US IDE Trial, which included a much larger patient cohort and enrolled patients from multiple sites [7].

## Conclusion

For Southeast Asian patients with RVOT conduit dysfunction or failed BPV, Melody TPVR provided hemodynamic and clinical improvements similar to those reported for patients in the US. In the majority of TPVR patients, a declining function was due to valve stenosis, but the need for reintervention remained low. A pre-stenting strategy was adopted, and no MSFs were observed. Postimplantation residual stenosis and progressive stenosis of the RVOT require long-term monitoring and reintervention. Lastly, IE remained a concern despite vigorous prevention and peri-procedural bacterial endocarditis prophylaxis.

## Data Availability

Study data can be obtained by sending a request to the corresponding author.

## Abbreviations

BPV: Bioprosthetic valve
CP: Cheatham Platinum
DORV: Double outlet right ventricle
IDE: Investigational Device Exemption
IE: Infective endocarditis
Kg: Kilogram
mm: Millimeter
MRI: Magnetic resonance imaging
MSFs: Melody valve stent fractures
PA: Pulmonary artery
PAVSD: Pulmonary atresia with ventricular septal defect
PR: Pulmonary regurgitation
PS: Pulmonary stenosis
RV: Right ventricle
RVOT: Right ventricular outflow tract
SD: Standard deviation
SE: Southeast
TOF: Tetralogy of Fallot
TPV: Transcatheter pulmonary valve
TPVR: Transcatheter pulmonary valve replacement
VF: Ventricular fibrillation
VSD: Ventricular septal defect
VT: Ventricular tachycardia
